# Handwriting but not typewriting leads to widespread brain connectivity: a high-density EEG study with implications for the classroom

**DOI:** 10.3389/fpsyg.2023.1219945

**Published:** 2024-01-26

**Authors:** F. R. (Ruud) Van der Weel, Audrey L. H. Van der Meer

**Affiliations:** Developmental Neuroscience Laboratory, Department of Psychology, Norwegian University of Science and Technology, Trondheim, Norway

**Keywords:** handwriting, typewriting, brain connectivity (coherence), high-density EEG, young adults (18–29 years)

## Abstract

As traditional handwriting is progressively being replaced by digital devices, it is essential to investigate the implications for the human brain. Brain electrical activity was recorded in 36 university students as they were handwriting visually presented words using a digital pen and typewriting the words on a keyboard. Connectivity analyses were performed on EEG data recorded with a 256-channel sensor array. When writing by hand, brain connectivity patterns were far more elaborate than when typewriting on a keyboard, as shown by widespread theta/alpha connectivity coherence patterns between network hubs and nodes in parietal and central brain regions. Existing literature indicates that connectivity patterns in these brain areas and at such frequencies are crucial for memory formation and for encoding new information and, therefore, are beneficial for learning. Our findings suggest that the spatiotemporal pattern from visual and proprioceptive information obtained through the precisely controlled hand movements when using a pen, contribute extensively to the brain’s connectivity patterns that promote learning. We urge that children, from an early age, must be exposed to handwriting activities in school to establish the neuronal connectivity patterns that provide the brain with optimal conditions for learning. Although it is vital to maintain handwriting practice at school, it is also important to keep up with continuously developing technological advances. Therefore, both teachers and students should be aware of which practice has the best learning effect in what context, for example when taking lecture notes or when writing an essay.

## Introduction

Digital devices are more and more replacing traditional handwriting (Longcamp et al., 2006; Kiefer et al., 2015), and as both writing and reading are becoming increasingly digitized in the classroom, we need to examine the implications of this practice (Mangen and Balsvik, 2016; Patterson and Patterson, 2017). Using a keyboard is now often recommended for young children as it is less demanding and frustrating (Cunningham and Stanovich, 1990; Fears and Lockman, 2018), allowing them to express themselves in written form earlier (Hultin and Westman, 2013). Be that as it may, handwriting training has not only been found to improve spelling accuracy (Cunningham and Stanovich, 1990) and better memory and recall (Longcamp et al., 2006; Smoker et al., 2009; Mueller and Oppenheimer, 2014), but also to facilitate letter recognition and understanding (Longcamp et al., 2005, 2008; Li and James, 2016). Such benefits for learning have been reported irrespective of when writing by hand using a traditional pen or pencil or using a digital pen (Osugi et al., 2019). Also, brain research shows that it is not just any motor activity that facilitates learning, but that accurately coordinating the complex hand movements while carefully shaping each letter when using a pen, is crucial (Pei et al., 2021). Apparently, the pen causes different underlying neurological processes that provide the brain with optimal conditions for learning and remembering (Askvik et al., 2020).

Recent findings in neuroscience reveal that neural processes are not as localized and static as is commonly believed, but that the brain is organized in a highly dynamic functional manner (Lopes da Silva, 1991; Singer, 1993). Under normal circumstances, several brain systems are continually working together (Buzsáki, 2006), showing an extremely flexible organization with structurally different neural tissue being involved in neural circuits that are only temporarily assembled so as to enable a given task (Edelman and Gally, 2013; Van der Weel et al., 2019). In such a view, neurons can change function entirely when incorporated in different systems (Anderson, 2014). Bullmore and Sporns (2009) refer to this type of flexible organization of the brain as functional connectivity as against structural connectivity.

Electroencephalography is well suited to studying brain electrical activity as a function of handwriting and typewriting in the millisecond scale. It permits the investigation of changes in the status of the underlying active networks (Lopes da Silva, 1991) and can reveal the everchanging spatial patterns of activations that are specific to any given task (Pfurtscheller et al., 1996). In particular, studies of cortical oscillations detected with high-density EEG are now considered an indispensable aspect of contemporary systems neuroscience (Fröhlich, 2016).

Brain oscillations can be considered as the interplay between the cortex and the thalamus and are generated by changes involved in the control of oscillations in neural networks (Pfurtscheller and Lopes da Silva, 1999). The complex interactions and the resulting particular frequencies are thought to reflect distinct cognitive processes (Klimesch et al., 1994; Berens and Horner, 2017). The temporal organization of neuronal firing is crucial as it is assumed to be fundamental when forming long-term memories in the hippocampus (Berens and Horner, 2017).

Frequency-specific changes in EEG recordings can be observed as event-related synchronization (ERS) or event-related desynchronization (ERD; Pfurtscheller and Aranibar, 1977; Pfurtscheller and Lopes da Silva, 1999). Spectral analyses are used to detect differences in a given frequency band (Pfurtscheller et al., 1994; Salmelin and Hari, 1994; Klimesch et al., 1996), by calculating the temporal dynamics of EEG oscillations and quantifying event-related amplifications and/or suppressions of rhythms.

A recent EEG-study from our lab showed that drawing by hand causes more activity and involves larger areas in the brain as opposed to typing on a keyboard (Van der Meer and Van der Weel, 2017). We concluded that the involvement of fine and intricate hand movements in notetaking, in contrast with pressing keys on a keyboard that all require the same simple finger movement, may be more advantageous for learning (Van der Meer and Van der Weel, 2017). A follow-up study observed event-related synchronized activity in the theta range in both children and students in parietal and central brain regions, but only when writing by hand (Askvik et al., 2020). As these studies have found evidence that writing by hand facilitates learning, the present study further investigated the neurobiological differences related to cursive writing and typewriting in the young adult brain. Specifically, we investigated how the various brain regions interconnect via neural networks when writing by hand as opposed to typing on a keyboard using frequency modulation and the latest in brain connectivity analysis (c.f., Solomon et al., 2017).

## Methods

### Participants

Forty university students in their early twenties took part in the study at the Developmental Neuroscience Laboratory, Norwegian University of Science and Technology (NTNU). HD EEG data from 36 students were of good enough quality and sufficiently artifact-free to be included in the analyses. The data from 12 adult participants were already used in analyses in the time-frequency domain (Askvik et al., 2020). The present study performed a brain connectivity analysis to investigate the underlying neural networks involved in tasks of handwriting and typewriting. Participants were mostly students and were recruited at the university campus. They received a $15 cinema ticket for taking part. To avoid crossover effects between the two hemispheres, only right-handed participants were included, as determined by the Edinburgh Handedness Inventory (Oldfield, 1971). Allowing the use of (the fingers of) both hands would cause many unforeseen effects on the brain, which would make it hard to interpret the results. Participants gave their informed written consent, and it was made clear that they could withdraw from the experiment at any time without consequences. The Regional Committee for Medical and Health Ethics (Central Norway) approved the study.

### Experimental stimuli and EEG data acquisition

E-prime 2.0 was used to individually display 15 different Pictionary words on a Microsoft Surface Studio. The participants used a digital pen to write in cursive by hand directly on the touchscreen, and a keyboard to typewrite the presented words.

**Figure 1:**
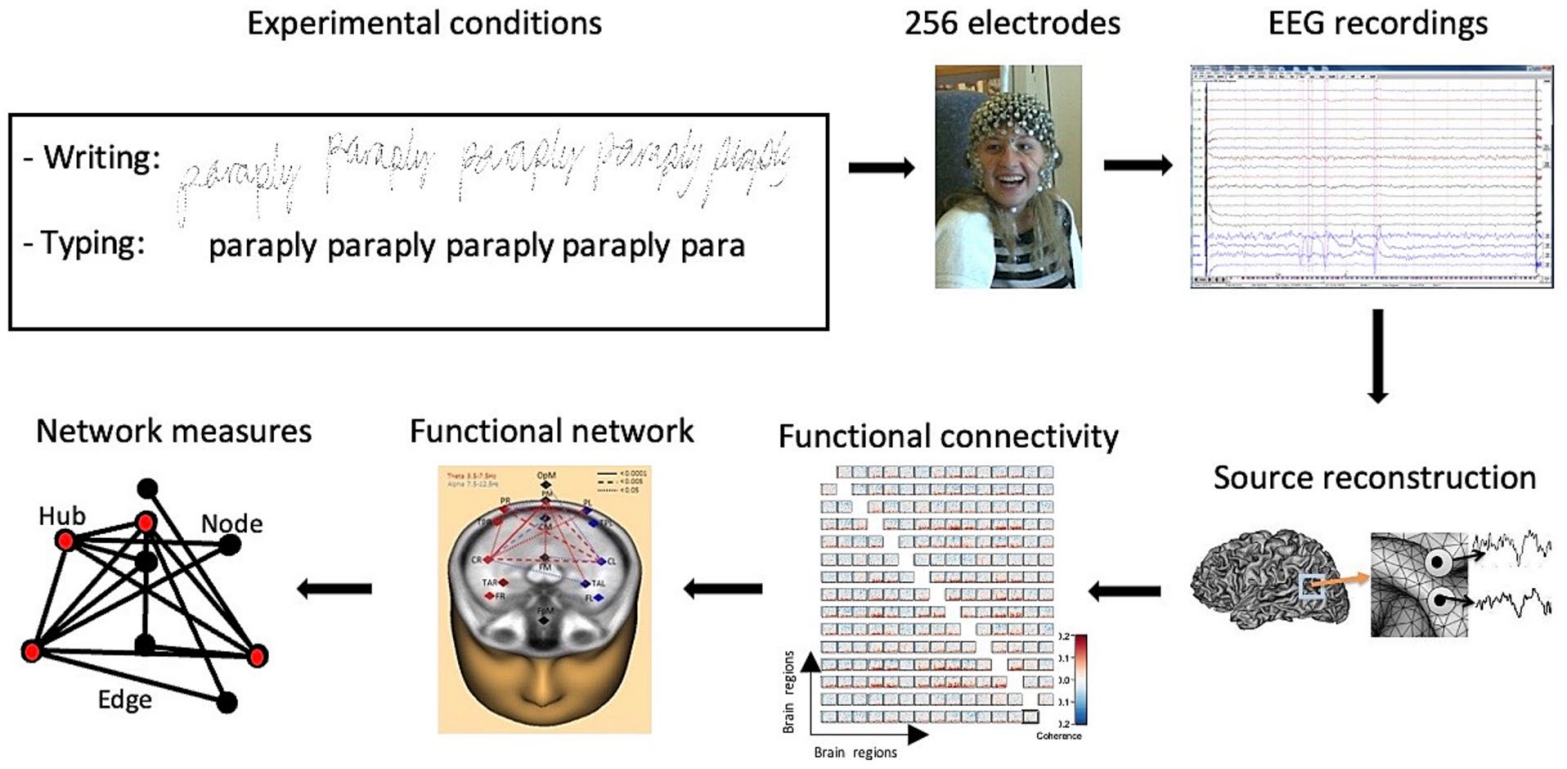
Task design, behavioral performance, and sequence of the connectivity analyses. Visually presented words were either written by hand with a digital pen or typed on a keyboard while participants were wearing a 256-channel sensor array. EEG recordings were analyzed in terms of their functional connectivity, resulting in detailed network measures.

The experiment comprised a total of 30 trials, where each word appeared in two different conditions, presented in a randomized order. For each trial, participants were instructed to either (a) *write in cursive* with their right hand the presented word with a digital pen directly on the screen, or (b) *type* the presented word using the right index finger on the keyboard. Before each trial, the instruction *write* or *type* appeared before one of the target words appeared, and the participants were given 25 s to either write by hand or type the word multiple times, separated by a space. EEG data were recorded only during the first 5 s of each trial. To prevent artifacts produced by head and eye movements caused by shifting gaze between the screen and the keyboard, typed words did not appear on the screen while the participant was typewriting. The writings produced by the participants (see Figure 1 for example) were stored for offline analyses.

A Geodesic Sensor Net (GSN; Tucker et al., 1994) with 256 evenly distributed electrodes was used to record EEG activity from the participant’s scalp at 500 Hz. The signals were amplified using a high-input EGI amplifier (Picton et al., 2000).

### Procedure

On arrival in the lab, a consent form with all necessary information was given to the participants to sign. While the participant completed the handedness test, an appropriately sized net was soaked in a saline electrolyte for 15 min to optimize electrical conductivity. The participant was sitting comfortably in an adjustable chair in front of a table. The screen was placed on the table as closely as possible to the participant. A keyboard was also placed in a preferred position for the participant, and a digital pen was used for writing on the touchscreen. A pre-test was completed before the experiment started, where one of the experimenters was present in the room with the participant.

### Brain data pre-analyses

Brain Electrical Source Analysis (BESA version 7.0) research software and BESA Connectivity (version 1.0) were used to analyze the EEG data. Epoch and filter settings were the same as in Askvik et al. (2020).

Channels contaminated by movement artifacts were either removed or interpolated using spherical spline interpolation (Perrin et al., 1989; Picton et al., 2000). Up to 10% of channels could be defined as bad. Artifact correction was applied using manual and semi-automatic artifact correction with fitting spatial filters (Berg and Scherg, 1994; Ille et al., 2002; Fujioka et al., 2011).

The mean number of accepted trials out of 15 was 14.1 (*SD* = 1.1) for handwriting and 13.3 (*SD* = 1.3) for typewriting. To analyze oscillatory brain activity, a time-frequency analysis in brain space was then performed on accepted trials, see Askvik et al. (2020) for details. Optimal separation of brain activity was achieved using source montages derived from a multiple source model where waveforms separated different brain activities (Scherg and Berg, 1991). Using this procedure, the time-frequency content of different brain regions can be separated even if their activities severely overlap at the surface of the scalp (Hoechstetter et al., 2004). Then, the connectivity measure of Coherence was applied, resulting in a symmetric connectivity matrix with the upper and lower triangular matrix showing pairwise clusters symmetrical to the diagonal.

### Statistical analyses

Probability of significance in connectivity values was tested with BESA Statistics 2.0, where connectivity measures for all participants were computed and the significant connectivity regions were used as guides in finding the extent of connectivity between the two experimental conditions of writing and typing. A combination of permutation tests and data clustering was employed. Permutation tests were applied to each set of time samples belonging to one frequency bin (Simes, 1986). Data clusters that showed a significant effect between conditions were assigned initial cluster values. Using within-group ANOVA’s, these initial cluster values were passed through permutation and assigned new clusters so that the significance of the initial cluster could be determined. A Bonferroni correction was used for multiple comparisons. As in Askvik et al. (2020), cluster alpha, the significance level for building clusters in time and/or frequency, was set at 0.01 and the number of permutations was set at 10.000. Low- and high cut-offs for frequency were kept at 2 Hz and 60 Hz respectively, and epochs were set from −250 to 4,500 ms.

## Results

High-density EEGs were recorded during the experimental handwriting and typing conditions. Artifacts were removed from the raw EEG recordings, then the inverse problem was solved by using a 4-shell ellipsoidal head model to analyze the brain regions of interest. The time series of the reconstructed sources were obtained and transformed into the frequency domain using complex demodulation. The functional connectivity between the reconstructed sources was computed using the coherence method. A high-resolution functional connectivity matrix was obtained, and the corresponding functional brain network was visualized. Network measures were then extracted from the network (Figure 1).

**Figure 2:**
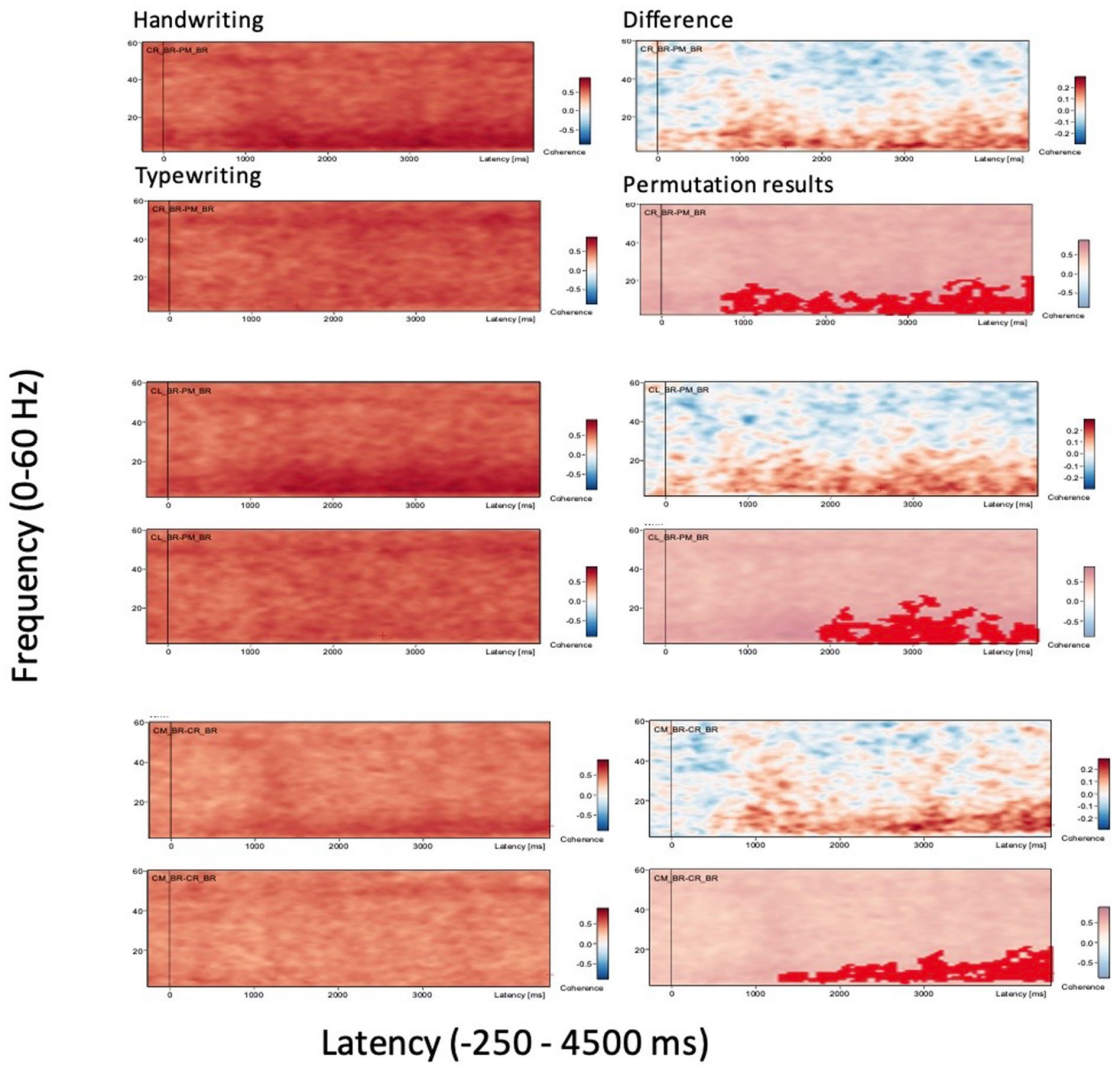
Grand average coherence results. Displayed are only three selected connectivity areas of interest for the two experimental conditions handwriting and typewriting (left panels), together with the difference in coherence between writing and typing and their permutation results (right panels). Connectivity areas of large significant difference between handwriting and typewriting included brain regions CR-PM (central right-parietal midline, top two panels on the left) and CL-PM (central left-parietal midline, middle two panels on the left), as well as CM-CR (central midline-central right, bottom two panels on the left), in frequencies ranging from theta (2 Hz) and up to gamma (60 Hz). The x-axes display the time interval from baseline to 4,500 ms of recordings of the trial. The signal magnitude reflects the estimated neural connectivity strength between the various brain areas during the experimental conditions compared to baseline activity (−250 to 0 ms). Positive connectivity is shown as (shades of) red-colored contours in handwriting/typewriting plots (panels on the left) and difference plots between handwriting and typewriting/permutation results (panels on the right). Positive connectivity is significantly more prominent in lower frequencies (theta 3.5–7.5 Hz and alpha 8–12.5) for handwriting (0 ≤ *p* < 0.05, see also Figure 4).

A time-frequency display is shown for three important brain regions in Figure 2 where the power/amplitude for each time is normalized to the mean power/amplitude of the baseline epoch for that frequency. The x-axis shows the time relative to the event, the y-axis shows the frequencies. The intensities are displayed as a color-coded plot.

Figure 2 displays the results of grand average coherence results from just three selected connectivity areas of interest for clarity, for the two experimental conditions handwriting and typewriting (left panels), together with the difference in coherence between writing and typing and their permutation results (right panels). Connectivity areas of large significant difference between writing and typing included central and parietal brain regions in frequencies ranging from theta (2 Hz) and up to gamma (60 Hz). The signal magnitude reflects estimated connectivity strength between brain areas compared to baseline (−250 to 0 ms) activity. Positive connectivity patterns are shown in (shades of) red. In the central and parietal areas, positive coherence patterns were more prominent in the lower frequencies (theta 3.5–7.5 Hz and alpha 7.5–12.5 Hz) for handwriting as opposed to typewriting. For handwriting, this activity appeared between 1,000 to 2000 ms and lasted throughout the trial.

### The connectivity matrix of writing over typing

**Figure 3:**
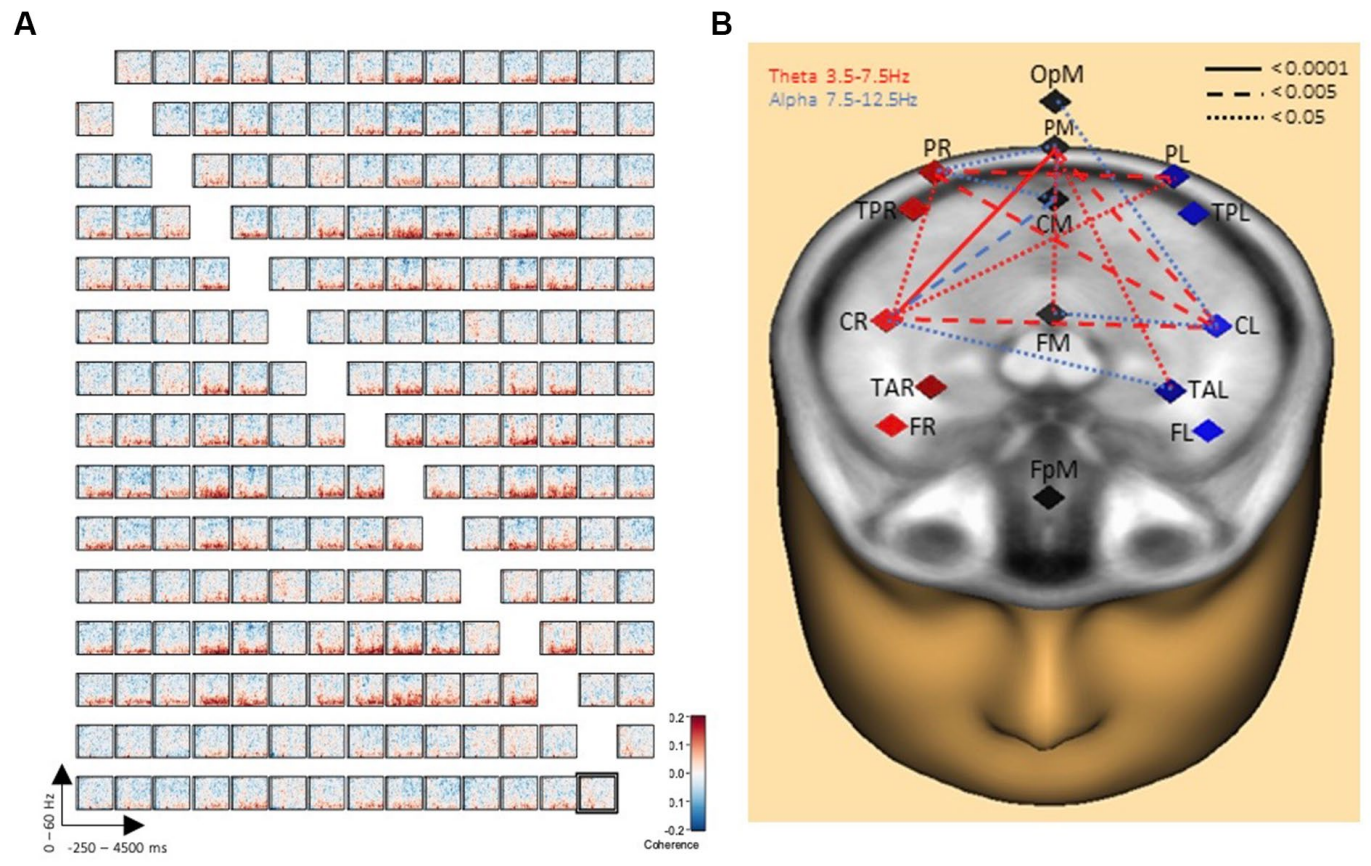
Connectivity results of writing over typing. **(A)** Grand average connectivity matrix results show widespread theta/alpha coherence results (in red) between PL, PM, PR and CL, CM, CR brain regions when writing by hand, but not when typing. The y-axes display frequencies from 2 to 60 Hz. The x-axes display the time interval from baseline to 4,500 ms of recordings of the trial for all involved brain regions. The signal magnitude (coherence) reflects the estimated neural connectivity between the various brain regions during the writing condition compared to baseline activity (−250 to 0 ms). **(B)** Further illustration of connectivity patterns revealing a concentration of 16 significant connections for handwriting compared to typewriting. Connection lines in red indicate connectivity in the theta range whereas lines in blue indicate connectivity in the alpha range. Levels of significance in connectivity strength for handwriting, but not for typewriting are further indicated by solid (<0.0001), dashed (<0.005), and dotted (<0.05) connection lines.

**Figure 4:**
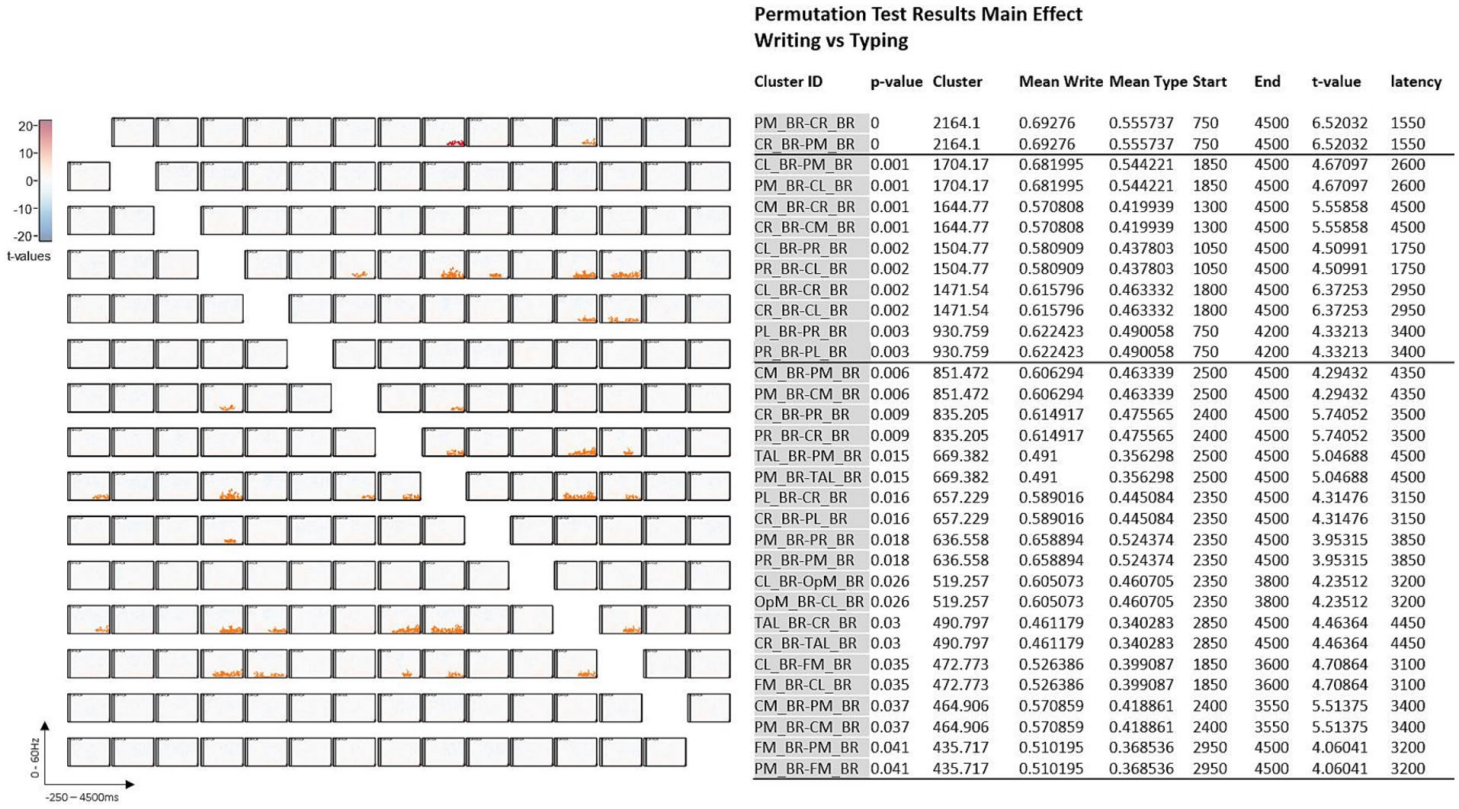
Symmetric connectivity matrix with *t*-values **(A)** and significance Table **(B)** with significant data clusters in the various sources of interest when handwriting is compared to typewriting in all participants. Thirty-two significant cluster differences marked in orange in **(A)** and fully described in **(B)** were found in the matrix and came out particularly significant in the parietal left (PL), parietal midline (PM), and parietal right (PR) areas.

Comparisons between the two conditions handwriting and typewriting were computed for each participant with time-frequency displays (changes in amplitude over time). TSE displays were limited between frequency cut-offs of 2–60 Hz, while frequency and time were sampled at 1 Hz and 50 ms, respectively. Symmetric connectivity measures were then obtained from BESA Connectivity and a high-resolution functional connectivity matrix between the reconstructed sources was computed using the coherence method (Rosenberg et al., 1989). Here, the number of in-phase components of two brain source signals at a specific frequency were described, and the corresponding functional brain network was visualized in Figure 3. Finally, network measures were extracted from the network and presented in Figure 4.

Figure 3A displays the grand average connectivity matrix for writing compared to typing. The matrix offers a compact description of the pairwise connectivity between all separate regions of the brain. Throughout the matrix there is evidence for widespread theta/alpha coherence results (in red) particularly between areas parietal-right, parietal-mid, and parietal-left and between areas central-right and central-left. These connectivity patterns are further illustrated in Figure 3B revealing a concentration of no less than 32 significant clusters (see Figure 4 for details) for handwriting, but not for typewriting. A pair of clusters will represent a single link between the corresponding pair of sources. The 32 significant clusters thus represent 16 significant connections.

### Main statistical effects

Analyses were run to test for statistical differences in brain activity between handwriting and typewriting. Figure 4 displays the detailed effects (*t*-tests) of the permutation results. These results showed 32 significant cluster differences between the two experimental conditions. The *t*-tests revealed significant differences in connectivity primarily in the theta (3.5–7.5 Hz) and alpha (8–12.5 Hz) range within three positive clusters (in orange), namely in the parietal left (PL), parietal midline (PM), and parietal right (PR) areas (see also Figure 3). These positive clusters suggest separate processes (differences in band power) between handwriting and typewriting mainly in the parietal but also in the central regions.

As can be seen in Figure 4, significant clusters of differences in band power were found mainly in parietal and central brain regions.

### Network measures

**Figure 5:**
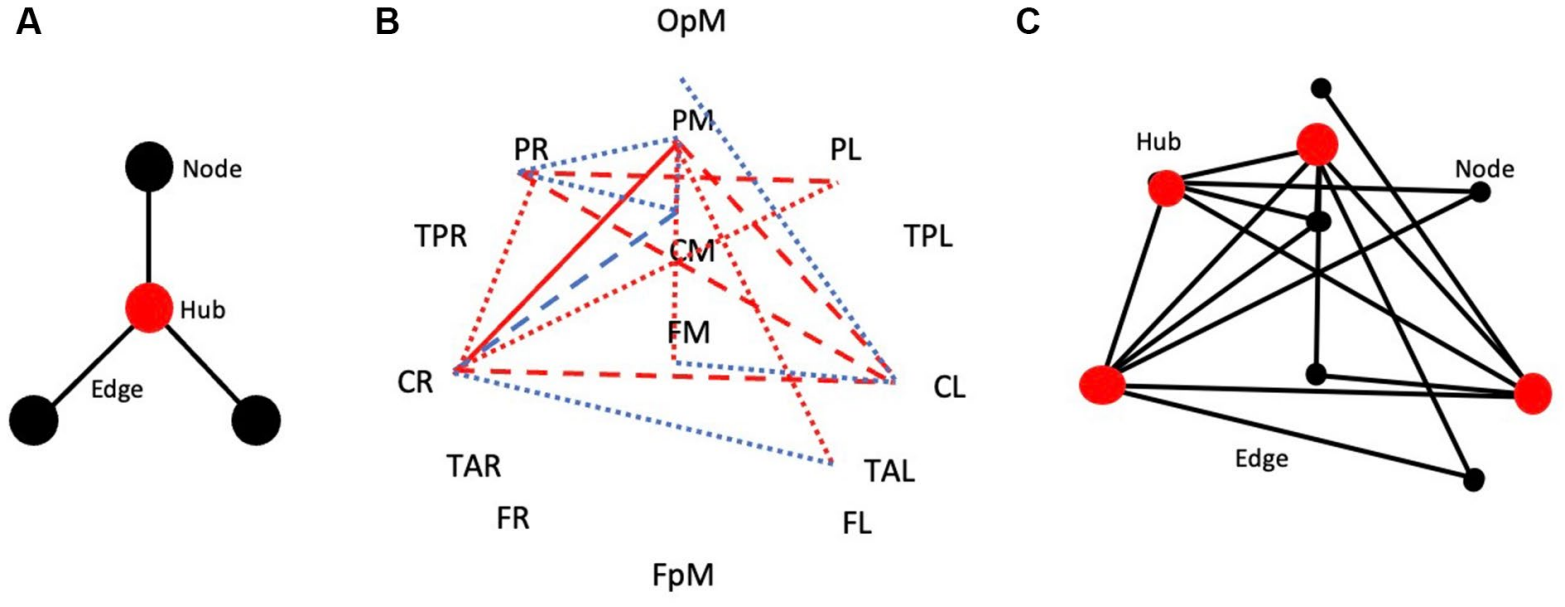
The adjacency matrix for handwriting. **(A)** Hub, nodes, and edges of a simplified theoretical network. **(B)** Brain connectivity network of handwriting compared to typewriting in this experiment. **(C)** Hubs (in red, ≥ 4 departures/arrivals) and nodes (in black, ≤ 3 departures/arrivals) interacting between brain regions PL, PM, PR and CL, CM, CR show widespread theta/alpha connectivity patterns when writing by hand, but not when typing.

Figure 5 shows the adjacency matrix for handwriting in the form of a hub, nodes, and edges of a simplified theoretical network (Figure 5A). Hubs have a higher degree of involvement in the network than nodes as expressed through their functional connectivity values (edges). Figure 5B shows the brain connectivity network results of handwriting compared to typewriting in this experiment. Proposed hubs (in red, ≥ 4 departures/arrivals) and nodes (in black, ≤ 3 departures/arrivals) interacting between brain regions PL, PM, PR and CL, CM, CR show widespread theta/alpha coherence patterns indicating stronger connectivity when writing as opposed to typing (Figure 5C).

## General discussion

This study investigated brain electrical connectivity as a function of handwriting and typewriting using high-density EEG in young adults. Participants used a digital pen to write visually presented words directly on a touchscreen and used a keyboard to type the words. Going beyond our previous study where we reported synchronized theta oscillations in parietal and central brain regions when children and students wrote by hand (Askvik et al., 2020), the present study performed connectivity analyses on the brain data of 36 students to explore underlying differences in coherence patterns when participants were typing versus writing by hand.

Focusing on brain connectivity that has shown to facilitate learning and memory (Pfurtscheller and Lopes da Silva, 1999), we investigated parietal and central areas in specific frequency bands. These brain areas have been associated with attentional mechanisms and cognitive processes in visual perception (Pfurtscheller et al., 1994; Vilhelmsen et al., 2019) and language (Brownsett and Wise, 2010; Benedek et al., 2014), and have strong links to sensorimotor cortex (Velasques et al., 2007). We set out to investigate whether it is actually the act of forming the letters by hand itself that brings about larger connectivity in the brain, since perceptual, motor, and higher cognitive areas are more involved during handwriting as opposed to typewriting.

### Increased connectivity in theta/alpha range for handwriting

The present findings revealed increased connectivity for handwriting over typewriting, suggesting that different underlying cognitive processes are involved in the two tasks. Increased connectivity within the theta (3.5–7.5 Hz) and alpha (8–12.5 Hz) frequency bands has been linked to mechanisms underlying sensorimotor integration (Bland and Oddie, 2001). As increased connectivity in the brain was observed only when writing by hand and not when simply pressing keys on the keyboard, our findings can be taken as evidence that handwriting promotes learning. Interestingly, the increased connectivity between the various brain regions seems to be linked to the specific sensorimotor processes that are so typical in handwriting.

The theta/alpha connectivity patterns found in the present study may indicate that different neural networks are involved in handwriting and typewriting. Interestingly, whereas connectivity in the alpha band is considered highly task-specific and is said to correspond to long-term memory performance, theta connectivity seems to be related to working memory and the ability to apprehend novel information (Klimesch et al., 1994, 1996, 2001; Klimesch, 1999; Raghavachari et al., 2001; Clouter et al., 2017). Thus, the enhanced brain connectivity for handwriting appears not to be related to differences in muscular involvement. It has also been proposed that hippocampal activity is reflected within the theta band (Klimesch et al., 1994), adding further support for the benefits of handwriting in terms of learning and memory formation.

Lower frequencies are considered especially suited for facilitating communication over longer distances in the brain, and are often reported to “gate” the occurrence of faster oscillations, for example when theta oscillations in humans are proposed to gate gamma (> 30 Hz) oscillations (Canolty et al., 2006; Halgren et al., 2018). In general, this theta-to-gamma cross-frequency coupling can be linked to gamma networks desynchronizing and theta networks synchronizing during encoding, retrieval, and episodic memory formation (Burke et al., 2013). Others have suggested that theta connectivity activity (see Figure 3) is positively correlated with a brain region’s gamma power, suggesting a potent low-frequency mechanism for communication between brain regions (Solomon et al., 2017). Exploring these interactions may disclose the relationship between a brain region’s functional connectivity and local processing. Our results reflect such a low-frequency mechanism for interregional communication. Present findings of theta synchrony for handwriting suggest that low-frequency connections support the integration of information during memory formation, and follow from earlier studies that have reported low-frequency entrainment to be essential to cognition (Solomon et al., 2017).

### The importance of handwriting practice in a learning environment

Handwriting requires fine motor control over the fingers, and it forces students to pay attention to what they are doing. Typing, on the other hand, requires mechanical and repetitive movements that trade awareness for speed. Our results reveal that whenever handwriting movements are included as a learning strategy, more of the brain gets stimulated, resulting in the formation of more complex neural network connectivity. It appears that the movements related to typewriting do not activate these connectivity networks the same way that handwriting does. The concurrent spatiotemporal pattern from vision, motor commands, and proprioceptive feedback provided through fine hand and finger movements, is lacking in typewriting, where only a simple key press is required to produce the entire wanted form (Longcamp et al., 2006; James, 2010; Vinci-Booher et al., 2016, 2021). In the present study, participants only used their right index finger for typing to prevent undesired crossover effects between the two hemispheres.

Thus, the ongoing substitution of handwriting by typewriting in almost every educational setting may seem somewhat misguided as it could affect the learning process in a negative way (Alonso, 2015; Mangen and Balsvik, 2016; Arnold et al., 2017). The present findings suggest that the intricate and precisely controlled handwriting movements have a beneficial impact on the brain’s connectivity patterns related to learning and remembering. The present study did not find evidence of such positive activation patterns when using a keyboard.

Even though maintaining handwriting practice in school is crucial, it is also important to keep up in the ever-developing digital world. Children should receive handwriting training at school to learn to write by hand successfully, and, at the same time learn to use a keyboard, depending on the task at hand. The present study shows that the neural connectivity patterns underlying handwriting and typewriting are distinctly different. Hence, being aware of when to write by hand or use a digital device is crucial, whether it is to take lecture notes to learn new concepts or to write longer essays.

## Data availability statement

The raw data supporting the conclusions of this article will be made available by the authors, without undue reservation.

## Ethics statement

The studies involving human participants were reviewed and approved by the Norwegian Data Protection Services for Research and by the Regional Committee for Medical and Health Ethics (Central Norway). The participants gave their written informed consent. Written informed consent was obtained from the individual(s) for the publication of any identifiable images or data included in this article.

## Author contributions

FW and AM contributed equally to all aspects of the study. All authors contributed to the article and approved the submitted version.
